# Stress and displacement pattern evaluation using two different palatal expanders in unilateral cleft lip and palate: a three-dimensional finite element analysis

**DOI:** 10.1186/s40510-016-0150-0

**Published:** 2016-11-21

**Authors:** Anoop Mathew, K. S. Nagachandran, Devaki Vijayalakshmi

**Affiliations:** 1 Department of Orthodontics, Indira Gandhi Institute of Dental Sciences, SBV University, Puducherry, 605402 India; 2 Department of Orthodontics and Dentofacial Orthopedics, Meenakshi Ammal Dental College & Hospital, Meenakshi University (MAHER), Alapakkam Main Road, Maduravoyal, Chennai, Tamilnadu 600095 India

**Keywords:** Finite element method, Rapid palatal expansion, Microimplant, Cleft lip and palate

## Abstract

**Background:**

In this finite element (FE) study, the stress distribution and displacement pattern was evaluated in the mid-palatal area and around circum-maxillary sutures exerted by bone-borne palatal expander (BBPE) in comparison with conventional HYRAX rapid palatal expander in unilateral cleft lip and palate.

**Methods:**

Computed tomography scan images of a patient with unilateral cleft palate was used to create a FE model of the maxillary bone along with circum-maxillary sutures. A three-dimensional model of the conventional HYRAX (Hygienic Rapid Expander) expander and custom-made BBPE was created by laser scanning and programmed into the FE model.

**Results:**

With the BBPE, the maximum stress was observed at the implant insertion site, whereas with the conventional HYRAX expander, it was at the dentition level. Among the circum-maxillary sutures, the zygomaticomaxillary suture experienced maximum stress followed by the zygomaticotemporal and nasomaxillary sutures. Displacement in the *X*-axis (transverse) was highest on the cleft side, and in the *Y*-axis (antero-posterior), it was highest in the posterior region in the BBPE.

**Conclusions:**

The total displacement was observed maximum in the mid-palatal cleft area in the BBPE, and it produced true skeletal expansion at the alveolar level without any dental tipping when compared with the conventional HYRAX expander.

## Background

Cleft lip and palate (CLP) is the most common craniofacial malformation characterized by underdeveloped maxilla both in transverse and sagittal dimension which can be corrected by surgical repair of the cleft followed by orthodontic treatment [1].

So far, the slow and rapid maxillary expansion appliances [2–10] that were tooth supported achieved expansion which were 50 % dental in nature [11, 12].

In recent years, bone-anchored rapid palatal expanders were developed with the advantage of directly anchoring the appliance to the palatal bone and many of them could achieve sufficient skeletal expansion without producing dental ill effects [13–19].

Several finite element (FE) analysis studies have been performed to assess the various biomechanical effects of rapid palatal expansion (RPE) in non-cleft patients [20–23]. Also, few studies have been reported analysing the stress pattern caused by conventional RPE appliances in CLP patients [24, 25]. Despite the varying conclusions that have been made in the literature regarding the differences between bone-anchored and tooth-borne rapid maxillary expansion, till date, no study has investigated the biomechanical effects of implant-supported RPE in cleft palate patients versus tooth-borne RPE.

Therefore, the purpose of this study was to evaluate the stress distribution and displacement pattern in the mid-palatal suture area and around circum-maxillary sutures using bone-borne palatal expander in a patient with unilateral cleft lip and palate in comparison with conventional HYRAX rapid maxillary expansion appliance using finite element analysis.

## Methods

### FEM model preparation

A computed tomographic scan of a 12-year-old patient with unilateral cleft lip and palate was obtained as a requisite for pre-treatment record which was approved by the institutional ethical committee. Then, the CT scan of the maxilla along with the teeth and circum-maxillary sutures was converted into a 3D model for finite element (FE) modelling.

**Fig. 1:**
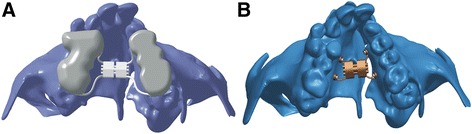
Three-dimensional model of **a** conventional HYRAX expander and **b** bone-borne palatal expander

The conventional HYRAX expansion screw (Leone, Italy) was laser scanned using a white light 3D scanner (Model: SmartScan 3D 2275, Breuckmann GmbH), and a 3D model was created (Fig. 1a). This expansion screw was activated to bring about 0.25-mm widening per turn, and twice a day activation protocol was followed.

Then, a custom-fabricated bone-borne palatal expander (BBPE) was constructed which consisted of a modified HYRAX expander (Leone, Italy) which enables microimplant placement and was laser scanned to create a 3D model (Fig. 1b).

In this study, C-implant (C-implant Co., Seoul, Korea) with a 1.8-mm diameter and 8.5-mm length was used and four implants were positioned in the FE model on the palatal slope: two between the canines and first pre-molars and two between the second pre-molars and first molars on both the left and right sides. The placement site for the microimplant was chosen based on the extent of the cleft and availability of the bone verified with the CT scan data within the biological limits without affecting the principles of design of the palatal expansion screw.

### Material properties

The material properties were assigned to the various structures such as the teeth, cancellous and compact bone and stainless steel in the FE model which are given in Table 1 and are in confirmation with the data available from previous studies [20–22, 26, 27].

**Table 1:** Material properties and elements used in the present study

S. no	Structures	Elements and nodes	Young’s modulus (MPa)	Poisson’s ratio
1	Cortical bone	Tetrahedral	13,700	0.30
2	Cancellous bone	Tetrahedral	7900	0.30
3	Sutures	Tetrahedral	10	0.49
4	Teeth	Tetrahedral	20,000	0.30

### Boundary conditions and solution

The process of meshing was carried out using pre-processor software Altair HyperMesh (Version 7.0), and specific boundary conditions were applied. Finally, the 3D FE model created consisted of 255,140 tetrahedral elements and 255,270 nodes.

The FE model was imported into ANSYS software (version 14.5), and various considerations are established. The laser-scanned conventional HYRAX expander and bone-borne palatal expander was attached onto the FE model. About 5-N force was applied to the model simulating the clinical situation, and analysis was performed which has been reported previously [24].

Analysis was done using a Newton–Raphson method of solving which efficiently handles non-linear problems accurately.

## Results

### Stress distribution in the mid-palatal area and circum-maxillary sutures in cleft palate

**Fig. 2:**
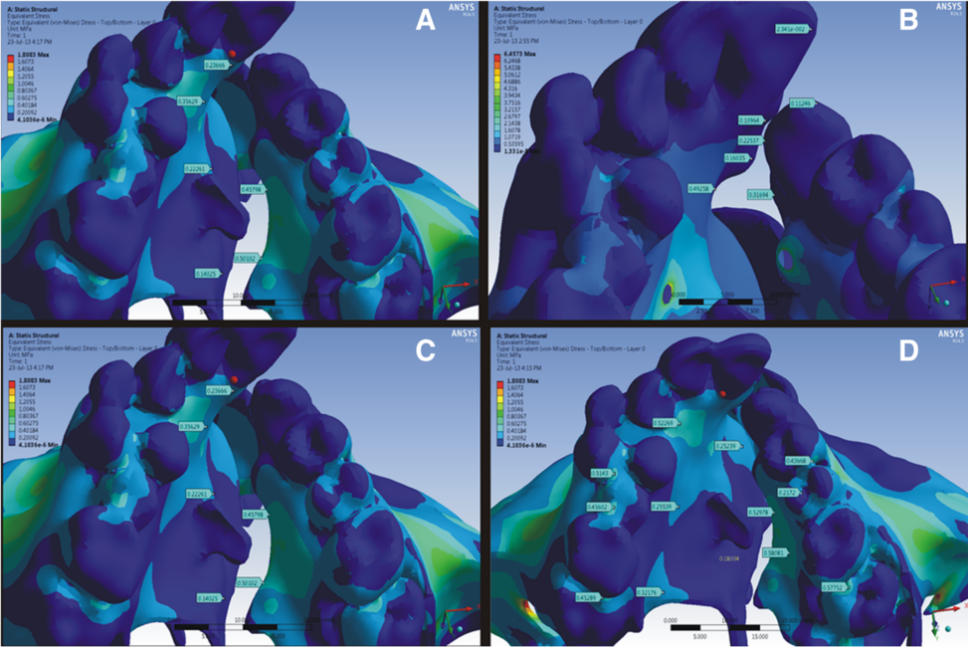
Von Mises stress distribution for the bone-borne HYRAX expander around the **a** mid-palatal suture area, **b** pre-maxillary region, **c** left palatal slope and **d** right palatal slope

**Fig. 3:**
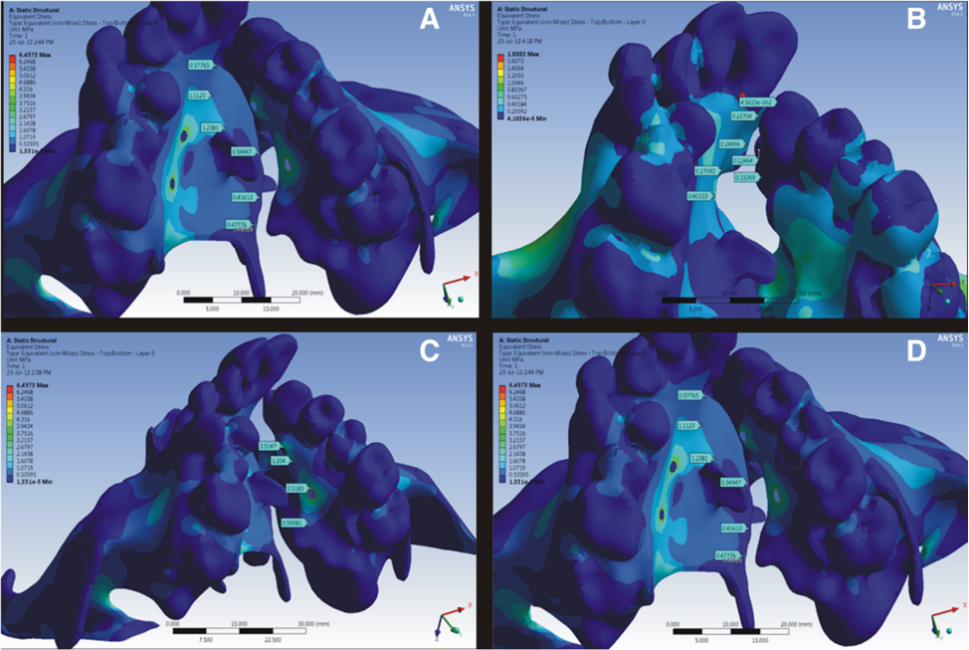
Von Mises stress distribution for the conventional HYRAX expander around the **a** mid-palatal suture area, **b** pre-maxillary region, **c** left palatal slope and **d** right palatal slope

In this study, with the bone-borne palatal expander, a greater amount of stress (4.320 MPa) was seen at the implant insertion site (Fig. 3). Also, the stress experienced in the primary palate cleft area assessed in the canine region was 0.8507 MPa, and the left and right sides of the cleft in the pre-maxillary region were 0.31694 and 0.1096 MPa, respectively. This is greater than that of the conventional HYRAX expander (Fig. 2). The comparison of stress created by the two appliances is shown in Table 2.

**Table 2:** Stress distribution (MPa) around the sutures and the cleft area

S. no	Sutures	Areas		Bone-borne palatal expander	Conventional HYRAX
1	Pre-maxilla cleft area	Non-cleft side		0.1096	0.2499
		Cleft side		0.31694	0.12464
2	Mid-palatal cleft area	Molars	Non-cleft side	0.6899	0.1835
			Cleft side	0.6824	0.4234
		Pre-molars	Non-cleft side	0.6204	0.2839
			Cleft side	0.6144	0.1043
		Canine	Non-cleft side	0.8507	0.4001
			Cleft side	0.7868	0.0779
3	Zygomaticomaxillary suture	Non-cleft side		0.56649	0.34412
		Cleft side		0.6079	0.6046
4	Zygomaticotemporal suture	Non-cleft side		0.7419	0.6104
		Cleft side		0.0855	0.10168
5	Nasomaxillary suture	Non-cleft side		0.69559	0.50771
		Cleft side		0.2343	0.33451

Similarly, in the secondary palate, the stress values in the pre-molar and first molar area were greater than that of the HYRAX expander (Fig. 3).

**Fig. 4:**
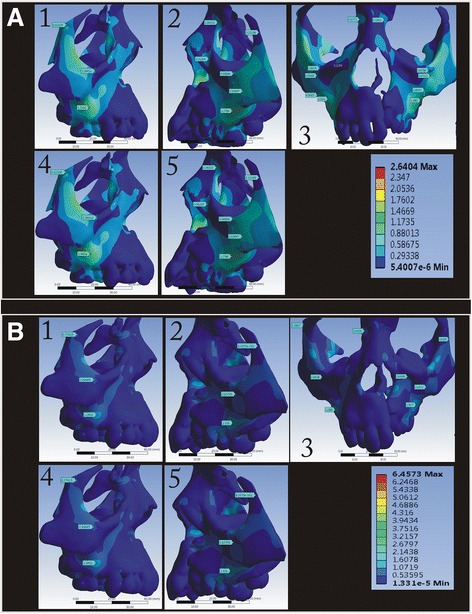
Von Mises stress fields around the circum-maxillary sutures in the **a** conventional HYRAX expander and **b** bone-borne HYRAX expander (**a1**, **b1**, zygomaticomaxillary suture non-cleft side; **a2**, **b2**, zygomaticomaxillary suture cleft side; **a3**, **b3**, nasomaxillary suture; **a4**, **b4**, zygomaticotemporal suture non-cleft side; and **a5**, **b5**, zygomaticotemporal suture cleft side)

In the circum-maxillary sutures, the highest stress was observed in the zygomaticomaxillary suture area (0.6079 MPa) which was followed by the zygomaticotemporal (0.0855 MPa) and nasomaxillary (0.2343 MPa) sutures in the BBPE when compared to the HYRAX expander (Fig. 4).

### Displacement pattern for the BBPE in comparison with the conventional HYRAX palatal expander

**Fig. 5:**
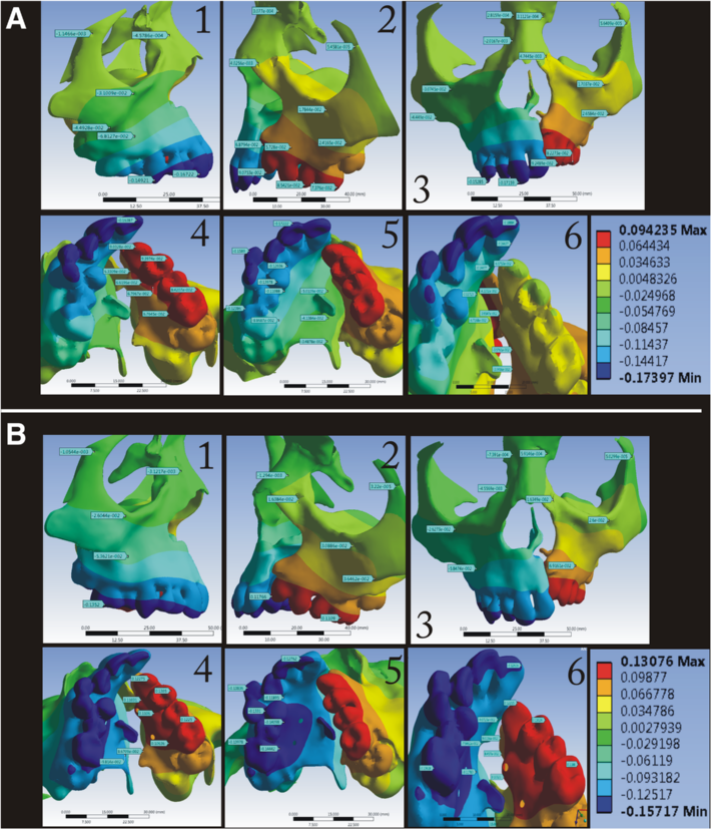
Displacement pattern in the *X*-axis for the **a** conventional HYRAX expander and **b** bone-borne HYRAX expander (**a1**, **b1**, non-cleft side; **a2**, **b2**, cleft side; **a3**, **b3**, frontal view; **a4**, **b4**, left palatal slope; **a5**, **b5**, right palatal slope; and **a6**, **b6**, mid-palatal area)

In the *X*-axis (transverse), maximum displacement was observed on the cleft side for both expanders (Fig. 5). In the BBPE, the displacement pattern was evenly distributed from the palatal slope to the dentition, whereas in the conventional expander, the displacement was concentrated maximum at the dentition level.

**Fig. 6:**
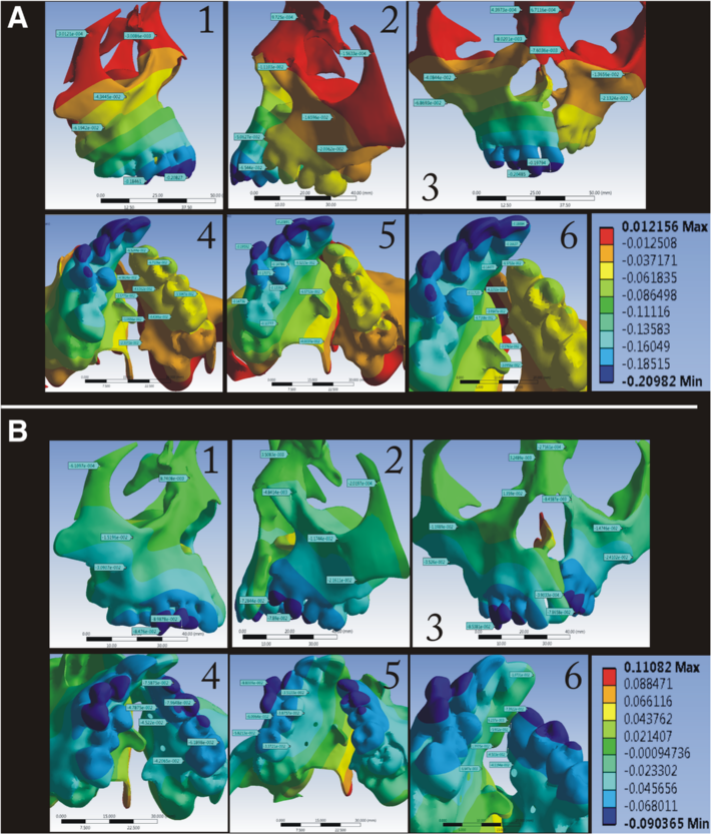
Displacement pattern in the *Y*-axis for the **a** conventional HYRAX expander and **b** bone-borne HYRAX expander (**a1**, **b1**, non-cleft side; **a2**, **b2**, cleft side; **a3**, **b3**, frontal view; **a4**, **b4**, left palatal slope; **a5**, **b5**, right palatal slope; and **a6**, **b6**, mid-palatal area)

In the *Y*-axis (antero-posterior), the maximum amount of displacement was observed in the posterior area, which was evenly extending anteriorly along the mid-palatal suture area in the BBPE, whereas in the conventional expander, the maximum displacement was seen in the cleft side concentrated more at the dentition level (Fig. 6).

**Fig. 7:**
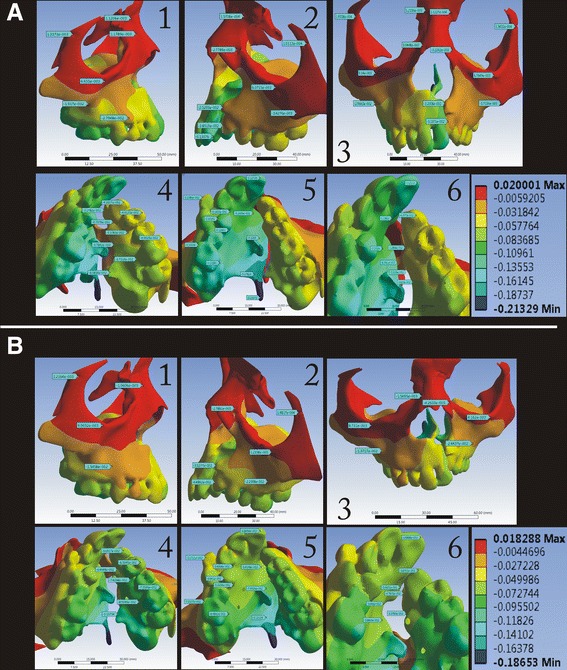
Displacement pattern in the *Z*-axis for the **a** conventional HYRAX expander and **b** bone-borne HYRAX expander (**a1**, **b1**, non-cleft side; **a2**, **b2**, cleft side; **a3**, **b3**, frontal view; **a4**, **b4**, left palatal slope; **a5**, **b5**, right palatal slope; and **a6**, **b6**, mid-palatal area)

In the *Z*-axis (vertical), the displacement was highest superiorly on both the cleft and non-cleft sides in the BBPE when compared to the conventional HYRAX expander where the displacement was maximum superiorly but more on the cleft side (Fig. 7).

Overall in all the axes, the maximum amount of displacement was seen along the mid-palatal suture area in the canine, pre-molar and molar regions with the BBPE when compared to the conventional HYRAX expander which is given in Tables 3 and 4.

**Table 3:** Displacement (mm) at the alveolar level in all three axes

Alveolar level	Conventional HYRAX rapid maxillary expander						Bone-borne palatal expander					
	Cleft side			Non-cleft side			Cleft side			Non-cleft side		
	*X*-axis	*Y*-axis	*Z*-axis	*X*-axis	*Y*-axis	*Z*-axis	*X*-axis	*Y*-axis	*Z*-axis	*X*-axis	*Y*-axis	*Z*-axis
Canine region	0.06330	0.04582	0.04773	0.01203	0.01447	0.09143	0.11031	0.04787	0.06464	0.01189	0.03510	0.05406
Pre-molar region	0.06619	0.04656	0.05108	0.01108	0.01339	0.01260	0.1106	0.04522	0.07426	0.01409	0.03875	0.06320
Molar region	0.06764	0.04434	0.06751	0.08868	0.01077	0.01338	0.10636	0.04206	0.08959	0.01444	0.03372	0.08486

**Table 4:** Displacement (mm) at the dentition level in all three axes

Dentition level	Conventional HYRAX rapid maxillary expander						Bone-borne palatal expander					
	Cleft side			Non-cleft side			Cleft side			Non-cleft side		
	*X*-axis	*Y*-axis	*Z*-axis	*X*-axis	*Y*-axis	*Z*-axis	*X*-axis	*Y*-axis	*Z*-axis	*X*-axis	*Y*-axis	*Z*-axis
Canine cusp tip	0.01589	0.06524	0.04360	0.09032	0.01959	0.05108	0.1227	0.07587	0.05691	0.01383	0.08800	0.03172
Pre-molar cusp tip	0.01397	0.06710	0.04412	0.09397	0.01707	0.01019	0.1305	0.07964	0.06504	0.01331	0.06006	0.06641
Molar cusp tip	0.01238	0.05594	0.04992	0.08420	0.01473	0.01053	0.1225	0.06189	0.07359	0.01397	0.05821	0.07001

## Discussion

RPE has been advocated for correcting maxillary transverse deficiency in cleft lip and palate followed by bone grafting [11]. Owing to the difficulty to achieve orthopaedic expansion with RPE alone, it is often combined with a surgically assisted procedure [12]. Recently, microimplant-anchored skeletal expansion can be a viable option which eliminates the need for surgical procedure [14–17].

Vyas et al. reported that the use of microimplant-assisted palatal distraction as an adjunct to SARPE provided adequate stable skeletal expansion [28]. Even though clinically they could achieve the skeletal expansion, the complex loading pattern and the biomechanical effects of this bone-borne palatal expander on the maxillary bone and the circum-maxillary sutures have not been evaluated. Hence, in the present study, we have used the bone-borne palatal expander and evaluated the stress distribution and displacement pattern using a finite element method.

The implants were placed on the palatal slopes to achieve efficient palatal expansion. Our results showed that with the BBPE, the greatest stress was seen at the implant insertion site which was gradually distributed along the palatal slopes on the cleft and non-cleft sides which is similar to the study reported by Lee et al. [29].

However, the stress distribution in the mid-palatal suture area was not clearly interpreted since there is absence of fusion of two palatal shelves in cleft palate. For this reason, we assessed the stress around the mid-palatal suture area in the pre-maxilla, canine, pre-molar and molar regions. With the BBPE, the greatest stress was observed around the pre-maxillary region as well as at the secondary palatal area when compared to the conventional HYRAX expander.

Holberg et al. in their finite element study has reported that RPE can produce up to 120 N of force and suggested that slow expanders with forces of about 5 N will suffice to bring about the necessary skeletal expansion in cleft patients [24, 30]. However, in the present study, we applied about 5 N of force in the FE model and evaluated the stress distribution around the cleft palate area and the circum-maxillary sutures.

Lee et al. reported that the maximum amount of orthodontic force that can be tolerated by the microimplant is about 400 g and with implants that are 1.8 mm in diameter, 400 g of orthodontic loading produces 30 MPa of force [31]. Furthermore, recent studies have reported that the mini-screws with an optimal 9-mm length had the ability to withstand about 2 N of force without breakage [32, 33]. However, in order to achieve orthopaedic maxillary expansion, it is necessary to apply 5 N of force which has been already reported by Holberg et al. [24], and hence, we applied 5 N of force in our study.

Moreover, due to certain limitations, we confined the force level to only 5 N and future studies might provide further information regarding the stress distribution produced by different force levels with this type of BBPE appliance and can predict ideal force levels and activation protocols.

Furthermore, the unilateral cleft maxilla FE model created by Holberg et al. [24] included about 30,138 tetrahedral elements and 55,064 nodes. In the present study, we have used 255,140 tetrahedral elements and 255,270 nodes to create a refined 3D unilateral cleft maxillary FE model. Studies have reported that the periodontal ligament of the teeth and the viscoelastic property when incorporated in a FE model will greatly influence the outcome of the stress distribution in a traditional tooth-borne rigid palatal expansion appliance [20, 34, 35]. Nonetheless, in the present study, the BBPE used is directly anchored to the bone but not to the teeth.

In the BBPE, transverse displacement was greater at the dentoalveolar region without buccal tipping of the teeth in the contrary conventional expander displaced at the dentition level. Hence, true skeletal expansion can be achieved in cleft palate with the BBPE since the forces are concentrated directly at the alveolar bone level. Nevertheless the bone available, the site of implant placement has to be considered as a pre-requisite, and the appliance design might vary in various cleft palate conditions before using the BBPE.

Among the circum-maxillary sutures, the zygomaticomaxillary suture on the cleft side experienced the highest stress with the BBPE when compared to the conventional HYRAX expander than on the non-cleft side. This agrees with the previous study reported by Gautam et al. [36, 37]. Several studies have also shown that the zygomatic buttress offers the primary resistance to different expansive forces in the circum-maxillary area [36–39]. Melsen and Melsen have reported that disarticulation is difficult in adolescents and adults due to heavy inter-digitations between the maxilla, palatine and pterygoid process of the sphenoid bone [40].

Stress at the zygomaticotemporal and nasomaxillary sutures on both the cleft and non-cleft sides in the BBPE was greater than that of the conventional HYRAX expander. However, the amount of stress experienced in these sutures was less than that of at the zygomaticomaxillary suture.

Recently, Ngan et al. had reported that in non-cleft class III individuals, the hybrid HYRAX bone-borne expansion device along with maxillary protraction yielded desirable sagittal skeletal change with minimal dental side effects [41]. Also Lin et al had concluded that in non-cleft individuals bone-borne maxillary expansion produced greater transverse orthopedic effects [42]. In such a case, if alternate expansion and constriction protocol is combined with BBPE, it might provide adequate disarticulation and future studies will define a refined expansion protocol in cleft palate patients.

Like many other finite element studies, this study without exception also has limitation due to the mathematical model as well as premises and assumptions used to generate the FE from a single patient which might not completely resemble the general population with individual variability as well as various clinical situations such as mid-palatal sutural viscoelastic property.

## Conclusions

- In the mid-palatal suture area, the greatest stress was observed at the implant insertion site on the cleft side along the palatal slopes and was evenly distributed superiorly to the alveolar and basal bone region with the use of the BBPE, whereas in the conventional HYRAX expander, the greatest stress was observed at the dentition level both on the cleft and non-cleft sides.
- Among the circum-maxillary sutures, the zygomaticomaxillary suture experienced the highest stress when using the BBPE which was followed by the zygomaticotemporal and nasomaxillary sutures when compared to the conventional HYRAX expander.
- The total displacement was observed maximum in the mid-palatal cleft area with the BBPE in comparison with the conventional HYRAX expander.
- Overall, the bone-borne palatal expander produced true skeletal expansion at the alveolar level without causing any dental tipping.
